# Soleus H-Reflex Inhibition Decreases During 30 s Static Stretching of Plantar Flexors, Showing Two Recovery Steps

**DOI:** 10.3389/fphys.2018.00935

**Published:** 2018-07-16

**Authors:** Francesco Budini, Monica Christova, Eugen Gallasch, Dietmar Rafolt, Markus Tilp

**Affiliations:** ^1^Institute of Sport Sciences, University of Graz, Graz, Austria; ^2^Institute of Physiotherapy, FH Joanneum – University of Applied Sciences, Graz, Austria; ^3^Otto Loewi Research Center, Physiology Section, Medical University of Graz, Graz, Austria; ^4^Center for Medical Physics and Biomedical Engineering, Medical University of Vienna, Vienna, Austria

**Keywords:** H-reflex, static stretching, post-activation depression, spinal excitability, ankle dorsiflexion, synaptic inhibitions

## Abstract

During the period when the ankle joint is kept in a dorsiflexed position, the soleus (SOL) H-reflex is inhibited. The nature of this inhibition is not fully understood. One hypothesis is that the decrease in spinal excitability could be attributed to post-activation depression of muscle spindle afferents due to their higher firing rate during the stretch-and-hold procedure. As the static stretching position is maintained though, a partial restoration of the neurotransmitter is expected and should mirror a decrease in H-reflex inhibition. In the present study, we explored the time course of spinal excitability during a period of stretching. SOL H-reflex was elicited during a passive dorsiflexion movement, at 3, 6, 9, 12, 18, 21, and 25 s during maximal ankle dorsiflexion, during plantar flexion (PF) and after stretching, in 12 healthy young individuals. Measurements during passive dorsiflexion, PF and after stretching were all performed with the ankle at 100° angle; measurements during static stretching were performed at individual maximal dorsiflexion. H-reflex was strongly inhibited during the dorsiflexion movement and at maximal dorsiflexion (*p* < 0.0001) but recovered during PF and after stretching. During stretching H-reflex showed a recovery pattern (*r* = 0.836, *P* = 0.019) with two distinct recovery steps at 6 and 21 s into stretching. It is hypothesized that the H-reflex inhibition observed until 18 s into stretching is the result of post-activation depression of Ia afferent caused by the passive dorsiflexion movement needed to move the ankle into testing position. From 21 s into stretching, the lower inhibition could be caused by a weaker post-activation depression, inhibition from secondary afferents or post-synaptic inhibitions.

## Introduction

During a passive lengthening movement of ankle plantar flexor muscles a decrease in H-reflex can be observed (Mark et al., 1968; Delwaide and Hugon, 1969; Burke et al., 1983; Romano and Schieppati, 1987; Vujnovich and Dawson, 1994; Nielsen et al., 1995; Hultborn et al., 1996; Voigt and Sinkjær, 1998; Pinniger et al., 2001; Nordlund et al., 2002; Avela et al., 2004; Duclay and Martin, 2005

; Robertson et al., 2012; Grosprêtre et al., 2014). This commonly detected phenomenon is generally attributed to presynaptic inhibitions (Mark et al., 1968; Romano and Schieppati, 1987; Nielsen et al., 1995; Voigt and Sinkjær, 1998). H-reflex is also inhibited throughout the time when the muscle is kept at the static end position of a stretch (Robinson et al., 1982; Etnyre and Abraham, 1986; Guissard et al., 1988, 2001; Vujnovich and Dawson, 1994; Hwang, 2002; Masugi et al., 2017). In this case though, presynaptic inhibitions having a short duration (Eccles et al., 1962; Kohn et al., 1997), it has been suggested that the decrease in spinal excitability could be attributed to reduced transmitter release from Ia afferents (Nielsen et al., 1995; Hultborn et al., 1996; Wood et al., 1996). However, H-reflex recovers immediately as the ankle joint is returned to its neutral position after a period of static stretching (Guissard et al., 1988, 2001; Vujnovich and Dawson, 1994; Yapicioglu et al., 2013; Opplert et al., 2016; Budini et al., 2018). This supports the hypothesis that the H-reflex goes through a recovery phase during the passive plantar flexion (PF) needed to reposition the foot to its neutral angle or during the application of the static stretch itself. In theory, if the reduction in spinal excitability is caused by post-activation depression when the muscle is kept in a static stretch position, the size of the H-reflex during static stretching should reflect the availability of neurotransmitter at Ia endings and indirectly reflect the afferent activity from muscle spindles. Therefore, neurotransmitter availability should decrease quickly during the passive dorsiflexion movement until reaching the static end position of the stretch when muscle spindles afferents activity is maximal (Matthews, 1933; Cooper, 1961). In combination with presynaptic inhibitions, this results in a considerable reduction in H-reflex size. Similarly, during a passive muscle shortening, since afferent firing of both type I and II fibers from muscle spindles is drastically reduced (Matthews, 1933; Cooper, 1961), neurotransmitter should be restored within few seconds and the H-reflex recover accordingly. In the static stretching position, the activity from muscle spindles afferents differentiate markedly: type II afferents is increased, whilst the activity of Ia afferents is much lower compared to the passive lengthening although still higher compared to rest condition and show a slow tendency to further decrease as the position is maintained (Matthews, 1933; Cooper, 1961). This reduction in firing rate should give the possibility to partially restore the neurotransmitter and should mirror a decrease in H-reflex inhibition. Therefore, by monitoring the time course of H-reflex inhibition during static stretching one could expect a recovery pattern.

The time course of H-reflex inhibition during static stretching has never been explicitly investigated. Previous studies (Robinson et al., 1982; Guissard et al., 1988) reported some results of H-reflex recovery during stretching, however, these are presented only marginally and without sufficient details and analysis to be able to draw any conclusion. Moreover, as highlighted in a recent review (Budini and Tilp, 2016), to be able to average an appropriate number of H-reflexes at the same time point during or following an intervention, the procedure has to be repeated several times. Otherwise, what one would be looking at is a mean value that does not represents the time course with appropriate time resolution.

The present study overcomes this methodological pitfall by repeating the stretching procedure several times and aims to monitor the soleus (SOL) H-reflex inhibition behavior during the entire period of static stretching of ankle plantar flexors. Additional aims were to monitor H-reflex values during dorsiflexion, PF, and after stretching.

## Materials and Methods

### Participants

Twelve healthy volunteers (24.1 ± 1.9 years, body mass 63.2 ± 9.3 kg, and stature 171.2 ± 10.2 cm) were recruited and required to abstain from any strenuous physical activity on the testing day. The study was approved by the University of Graz ethics board (GZ. 39/77/63 ex 2013/14) and written informed consent was obtained from all volunteers.

### Study Design

The experiment consisted in the measurement of H-reflex: during passive ankle dorsiflexion, at different time points during static stretching, during passive ankle PF, and following stretching. Eight H-reflexes stimulations were performed at each of 3, 6, 9, 12, 18, 21, and 25 s into stretching, respectively. Fourteen H-reflexes were elicited during dorsiflexion, and 16 during PF as well as 90 s after stretching, respectively.

### Experimental Procedures

Subjects sat on an isokinetic dynamometer (CON-TREX MJ, Dübendorf, Switzerland) with the trunk at 110°, the right knee fully extended and the foot resting on the dynamometer footplate with the ankle angle set at 100° (10° PF deviating from a neutral position at 90°). By using a remote control, the volunteers were instructed to adjust the dorsiflexion isokinetic rotation operated by the dynamometer around the foot plate until the point of perceived maximal dorsiflexion. Participants were asked to keep their knee extended and to relax during the procedures.

**FIGURE 1:**
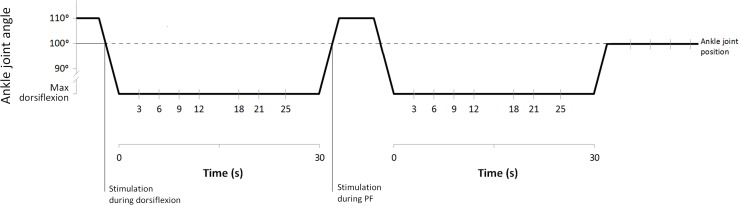
Stretching protocol and stimulation points.

Once the maximal individual dorsiflexion was defined, subjects left the dynamometer and were prepared for electromyographic (EMG) recording from SOL muscle. Subsequently, the volunteers sat down again on the dynamometer chair (position described above) and were then instructed to relax meanwhile two complete H-M stimulation ramps and 15 H-reflexes at about 5% M_max_ for baseline reference were collected. During the experiment, stimulations were delivered either with the ankle at 100° (during PF, during dorsiflexion, and after stretching) or at maximal individual dorsiflexion (during stretching) (**Figure 1**). To do this, following the baseline recordings, the foot was initially passively plantar flexed to a starting position of 110°. In this way, the stimulations during dorsiflexion movements (20°/s) could be delivered when the ankle reached 100°. Once individual maximal dorsiflexion was reached, the position was kept for 30 s, during this period, one H-reflex was delivered at one of the investigated time points (3, 6, 9, 12, 18, 21, and 25 s). After static stretching the foot was passively plantar flexed (20°/s) back to 110° and a stimulation was delivered during the PF movement when the ankle reached 100°. The procedure was repeated a second time with stimulations delivered only at maximal dorsiflexion, following stretching the foot was passively rotated to 100° for post-stretching measurements (**Figure 1**). This was repeated 30 times with randomly varying measurement time points in order to collect a sufficient number of H-reflexes at the desired time points.

### Stimulations

Electrodes (Blue Sensor N, Ambu A/S, Ballerup, Denmark) for recording H-reflex from the SOL muscle were placed in monopolar configuration (as suggested by Hadoush et al., 2009); the gain for the EMG signal was 180. All stimulations at baseline (15 H-reflex and 2 H-M ramps), during dorsiflexion and PF and after stretching were performed with the ankle joint at 100° (**Figure 1**). Stimulations during stretching were performed at individual maximal dorsiflexion position. H and M-waves measured in SOL were elicited by electrical stimulation (KeyPoint^®^ 2-channel) delivered to the tibial nerve by rectangular pulses of 1.0 ms duration. The anode (5 cm × 9 cm; STIMEX adhesive gel electrode) was placed on the patellar tendon, and the cathode was placed in the popliteal fossa overlying the nerve at a position that provided the greatest H-wave amplitude at the smallest stimulus intensity possible. The stimulation intensity used throughout the experiment was set to a value at which the H-wave was still in its ascending phase and an M-wave was visible (this stimulation intensity was usually close to the H_max_, and corresponded to about 5% of the M_max_).

### Data Analysis

Electromyography, foot displacement, and trigger signals were synchronized (DEWESoft^TM^ 7.0 recording system, DEWETRON GmbH, Austria), digitized with a sampling frequency of 10 kHz, stored on a PC. In order to avoid phase shift no low pass filter was applied. Limitation of the bandwidth with 60 kHz was determined by the isolation amplifier. No aliasing effect was observed. Data was analyzed using custom algorithms developed in Matlab (R2014b).

The H-reflexes recorded during stretching were checked for stimulation intensity consistency and those related to an M-wave showing peak to peak amplitude exceeding the average of the 56 M-waves (eight stimulations for each of the seven time points) by ±2 standard deviations were discarded (Budini et al., 2017). On average 5.3 H-waves were retained per measurement. Because the M-wave magnitude is altered during stretching (Tucker and Türker, 2007), the stimulation intensity during maximal dorsiflexion could not be compared to the one used at baseline. For this reason, for assessing the variation of the reflex during stretching, each H-wave was compared to the average of as many H-waves as possible induced with a similar stimulation intensity (±5% of the elicited M-wave amplitude) collected during the H-M ramps.

### Statistical Analysis

Data were checked for normal distribution by Shapiro–Wilk test.

ANOVA for repeated measures, was used to compare the level of H-reflex variation (expressed as percentage of control values) at the different time points during stretching. Time course of H-reflex during the stretching period was assessed with Pearson’s correlation.

Comparisons between the amplitude of the H-waves expressed as percentage of control waves, during dorsiflexion, stretching (average over the entire stretching time), and PF and post-stretching were assessed with an ANOVA for repeated measured and Bonferroni *post hoc* test.

Comparison between variations from baseline for dorsiflexion, PF and each stretching time point was assessed by paired *T*-test or Wilcoxon with Bonferroni adjustment.

Paired *T*-test was used to compare H-waves at each time point during stretching to the corresponding control H-waves collected during the recruitment ramps. H-waves at dorsiflexion, PF and after stretching were compared to the average H-waves at baseline through paired *T*-test.

## Results

### During Stretching

**FIGURE 2:**
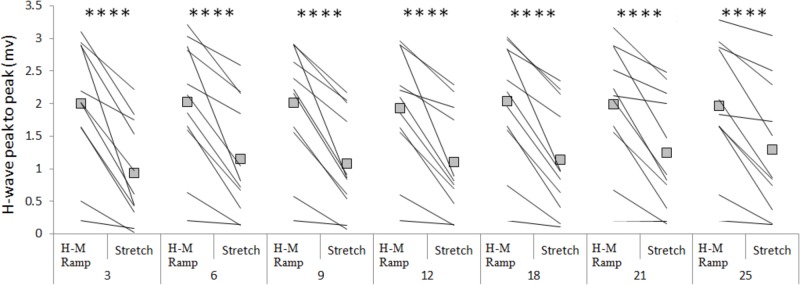
Group average (squares) and individual values (lines) for H-reflex peak to peak at control (H-M ramp) and at each tested time point during stretching. ^∗∗∗∗^*P* < 0.0001.

**FIGURE 3:**
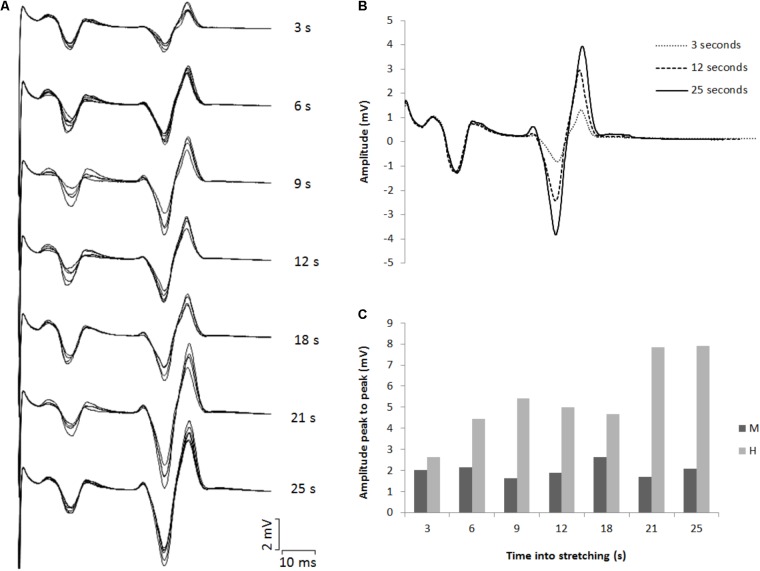
**(A)** Superimposed EMG tracks for each time point tested during stretching. **(B)** Three superimposed H-waves induced at 3, 12, and 25 s into stretching. **(C)** Average peak to peak amplitude for eight M- and H-waves for each time point during stretching.

**FIGURE 4:**
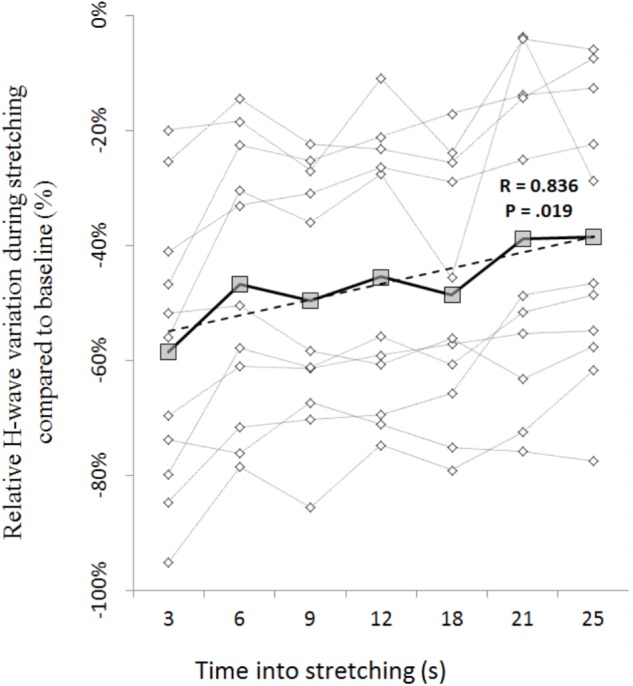
Time course of H-reflex inhibition during stretching for each participant (dotted lines with diamonds) and group average (solid line with squares). Group trend line is shown by the dashed line.

The amplitude of the H-waves collected at each time point during stretching was smaller than the amplitude of the H-waves elicited at similar stimulation intensity during the recruitment ramps (*P* < 0.0001 for all comparisons) (**Figure 2**). This was observed in all but one volunteer who was excluded due to inconsistency in stimulation intensity. As depicted from a representative subject in **Figure 3**, the inhibition was different between time points with a pattern to recover from 3 to 25 s into stretching. Group average values confirm both the differences between time points (*P* = 0.000, *F* = 8.737) and the recovery pattern (*r* = 0.836, *P* = 0.019) (**Figure 4**).

### During Dorsiflexion and Plantar Flexion

**FIGURE 5:**
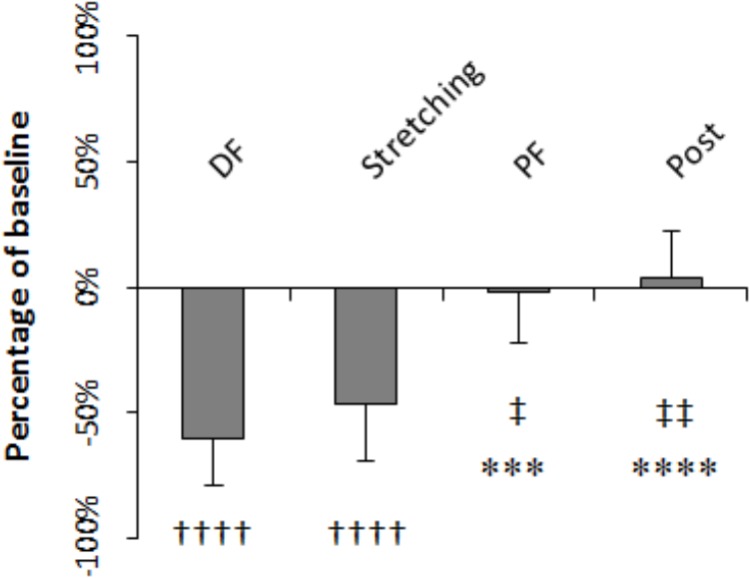
Amplitude expressed as percentage of control values. Comparison to control: ^††††^*P* < 0.0001; comparison to dorsiflexion: ^∗∗∗^*P* < 0.001 and ^∗∗∗∗^*P* < 0.0001; comparison to stretching: ^‡^*P* < 0.05 and ^‡‡^*P* < 0.01.

H-reflex was on average 60.4% smaller during dorsiflexion than at baseline (*P* = 0.000) whereas during PF and after stretching it did not differ significantly to baseline (-1.8%, *P* = 0.203 and +4%, *P* = 0.299, respectively). The level of H-reflex reduction observed during dorsiflexion, stretching (average of all the time points), PF, and post-stretching was different (*P* = 0.000, *F* = 28.208), with a higher inhibition during stretching and dorsiflexion compared to during PF (*P* = 0.041 and *P* = 0.005) and post-stretching (*P* = 0.001 and *P* = 0.000) (**Figure 5**). There were no differences between stretching and dorsiflexion (*P* = 0.187) and between PF and post-stretching (*P* = 0.108). When comparing H-reflexes during dorsiflexion and PF to each time point during stretching, following Bonferroni-Holmes correction, the inhibition during dorsiflexion was greater than the inhibition at time point 21 and 25 only (*P* = 0.005 and *P* = 0.004, respectively). Differently, H-reflex during PF was bigger than H-reflex at every time point during stretching (*P* < 0.05 for all comparison).

## Discussion

When a muscle is kept in an elongated position as during muscle stretching, a reduction in H-reflex is consistently reported (for review, see Budini and Tilp, 2016). Our result in this respect is therefore in agreement with literature. About the nature of this inhibition, mechanisms such as joint mechanoreceptors and skin receptors (Robinson et al., 1982; Hultborn et al., 1996), classical pre-synaptic inhibition (Hultborn et al., 1996; Wood et al., 1996), and moto-neural excitability (Hultborn et al., 1996; Budini et al., 2017, 2018) have all been excluded. Moreover, activity from Ib afferents, that would normally feed inhibition to their homonymous muscles when tension is detected in the tendons, can also be discarded because Golgi organs are relatively insensitive to passive stretching (Burg et al., 1973; Jami, 1992). Most of the authors therefore support the hypothesis that the reduced size of the H-wave is to be attributed to reduced transmitter release from Ia afferents (post-activation depression) (Robinson et al., 1982; Nielsen et al., 1995; Hultborn et al., 1996; Wood et al., 1996).

**FIGURE 6:**
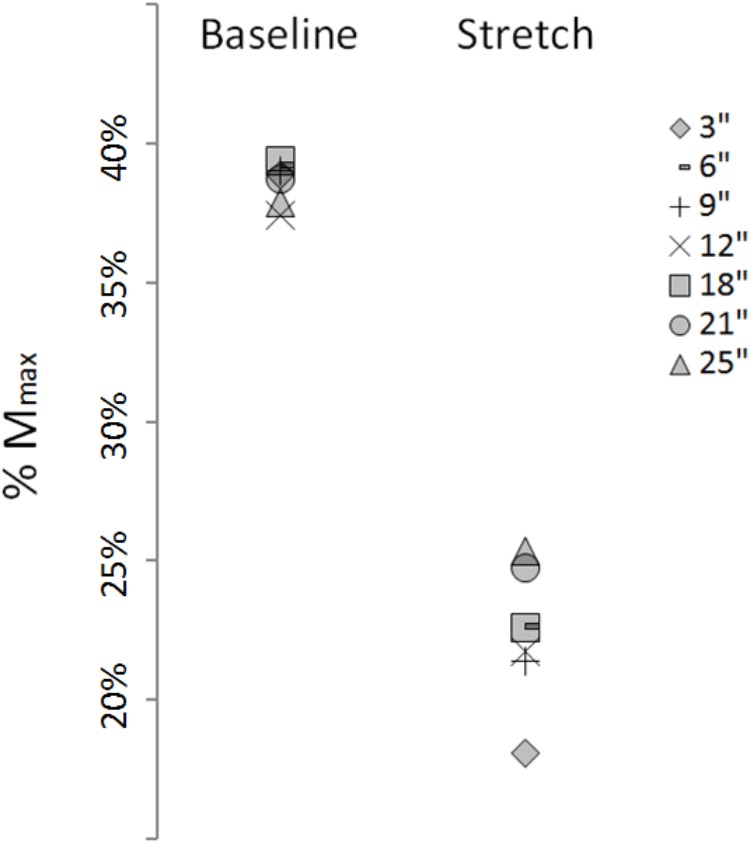
Group average H-waves expressed as percentage of M_max_ at baseline and during stretching.

The time course of the H-reflex inhibition (**Figure 4**) provides sustaining evidence to the post-activation depression hypothesis. Indeed, as we anticipated, the reflex recovered during the stretching time and this can be attributed to a replenishment of neurotransmitter in response to reduced Ia afferent activity. Type I fibers from muscle spindles have a basal firing rate which increases during elongation of the spindle which quickly decrease at the end of the elongation and then further slowly decrease as the stretch is maintained (Matthews, 1933; Cooper, 1961). As the firing frequency decreases, more transmitter becomes again available. Interestingly though, the recovery phase we observed was not linear. As it can be observed from the representative subject in **Figure 3**, the inhibition was larger at 3 s into stretching, plateaued between 6 and 18 s for then start recovering again from 21 s. This result was confirmed in almost every participant (**Figures 4, 6**).

These recovery steps reflect the spindle behavior (for review, see Hulliger, 1984) and can be attributed to the much higher Ia firing rate during the dorsiflexion movement that caused a stronger reduction of neurotransmitter release for several seconds. Therefore the inhibition at 3 s into stretching should be considered more as a protracted effect of the passive dorsiflexion than the response to stretching itself. Similarly, the second recovery step (6–18 s) can be thought as a weaker long lasting effect of the dorsiflexion. Comparable results were reported after a single passive ankle dorsiflexion where the inhibition decreases drastically during the first 6 s for disappearing only after 15 s (Nielsen et al., 1993, 1995; Hultborn et al., 1996; Wood et al., 1996; Voigt and Sinkjær, 1998). In agreement, in our study, the H-reflex reduction during dorsiflexion was not different to the inhibition during stretching until 21 s into stretching. In this perspective, the inhibition induced by the static stretching alone should be considered the one starting 21 s into stretching. Not having further time points after 25 s we do not know whether a new plateau or a new recovery step will follow.

The nature of the H-reflex inhibition from 21 s into stretching remains unclear. We excluded the possibility that this could be attributed to a long lasting inhibiting effect caused by the passive dorsiflexion, but whether the inhibition is still caused by a weaker post-activation depression of Ia terminals or some other neuro mechanism cannot be distinguished with our protocol. Firing frequency of muscle spindle secondary afferents increase when a muscle is kept in elongated position (Matthews, 1933; Cooper, 1961) and could possibly have a role as inhibiting factors at presynaptic level. Alternatively, the depression of the H-reflex can be the consequence of post-synaptic inhibition as proposed by Guissard et al. (2001). Among the post-synaptic inhibitory mechanisms, a variation in recurrent inhibition by Renshaw cell, as already suggested (Guissard et al., 2001), could be considered.

### Study Limitations

A methodological requirement for the present study consisted in repeating the stretching procedure several times. This can raise the question whether some cumulative effects might have influenced our results. In a recent work (Budini et al., 2018), we adopted the same protocol and observed that H-reflex recovers immediately to baseline level after stretching even if static stretching bouts of 30 s are repeated many times. This result suggests that in the present study the neuromuscular parameters related to spinal excitability were likely reset after each stretching bout without cumulative effects.

## Conclusion

In conclusion, for the first time the course of H-reflex inhibition throughout a period of static stretching was thoroughly explored. H-reflex showed a greater inhibition within the first 3 s and a clear recovery at 6 s; from 6 to 18 s a plateau was observed followed by another recovery step at 21 s into stretching. This behavior resembles the response of muscle spindles to a stretch-and-hold procedure, which suggests that until 18 s the inhibition can be attributed to the passive DF movement with related increased activity from primary afferents and subsequent depletion of neurotransmitter. Starting from 21 s the inhibition can be attributed to static stretching and possibly caused by a weaker post-activation depression, inhibition from secondary afferents or post-synaptic inhibitions. Whatever the cause the H-reflex shows a complete recover during PF and no effects were observed after the procedure.

## Author Contributions

FB, EG, MC, DR, and MT conceived and designed the work. FB and EG acquired, analyzed, and interpreted the data for the work. FB, EG, and MT drafted the work and revised it critically for important intellectual content. All authors approved the final version to be published.

## Conflict of Interest Statement

The authors declare that the research was conducted in the absence of any commercial or financial relationships that could be construed as a potential conflict of interest.
